# Breastfeeding practice in China from 2013 to 2018: a study from a national dynamic follow-up surveillance

**DOI:** 10.1186/s12889-021-10211-2

**Published:** 2021-02-10

**Authors:** Leni Kang, Juan Liang, Chunhua He, Lei Miao, Xiaohong Li, Li Dai, Qi Li, Zheng Liu, Jun Zhu, Yanping Wang, Hanmin Liu

**Affiliations:** 1 National Office for Maternal and Child Health Surveillance, West China Second University Hospital, Sichuan University, Chengdu, China; 2 Key Laboratory of Birth Defects and Related Diseases of Women and Children of the Ministry of Education, West China Second University Hospital, Sichuan University, Chengdu, China; 3 Department of Pediatrics, West China Second University Hospital, Sichuan University, No. 17, Section 3, South Renmin Road, Chengdu, 610041 China

**Keywords:** Early initiation of breastfeeding, Exclusive breastfeeding, Trend, Geographic disparities, Dynamic surveillance

## Abstract

**Background:**

Breastfeeding is important for the physical and psychological health of the mother and child. Basic data on breastfeeding practice in China are out-of-date and vary widely. This study aimed to evaluate the progress of breastfeeding practice in China, as well as to explore the bottlenecks in driving better practice.

**Methods:**

This was an observational study. We used data from the Under-5 Child Nutrition and Health Surveillance System in China for the period 2013–2018. The prevalence of early initiation of breastfeeding (EIBF) and exclusive breastfeeding (EBF) were calculated for each year for subgroups of China. The Cochran-Armitage test was used to explore the time trends. The annual percent of change (APC) were calculated by log-linear regression followed by *exp* transformation.

**Results:**

The prevalence of EIBF increased significantly from 44.57% (95% CI: 44.07, 45.07) in 2013 to 55.84% (95% CI: 55.29, 56.38) in 2018 (P_trend_ < 0.001), with an APC of 4.67% (95% CI: 3.51, 5.85). And the prevalence of EBF increased rapidly from 16.14% (95% CI: 15.10, 17.18) to 34.90% (95% CI: 33.54, 36.26) (P_trend_ < 0.001), with an APC of 14.90% (95% CI: 9.97, 20.04). Increases were observed in both urban and rural areas, with urban areas showing greater APCs for EIBF (6.05%; 95% CI: 4.22, 7.92 v.s. 2.26%; 95% CI: 1.40, 3.12) and EBF (18.21%; 95% CI: 11.53, 25.29 v.s. 9.43%; 95% CI: 5.52, 13.49). The highest EBF prevalence was observed in the East, but the Central area showed the highest APC. The prevalence of EBF decreased with increasing age within the first 6 months, especially after 3 months.

**Conclusion:**

The prevalence of both EIBF and EBF in China are improving in recent years. The rural and West China could be the key areas in the future actions. More efforts should be made to protect and promote breastfeeding to achieve near- and long-term goals for child health.

**Supplementary Information:**

The online version contains supplementary material available at 10.1186/s12889-021-10211-2.

## Background

Breastfeeding is important for the physical and psychological health of the mother and child. Scaling up breastfeeding could save the lives of 823,000 children in low- and middle-income countries every year [1]. Breastfeeding protects against a half of diarrhea morbidity and a third of respiratory infections [2], and reduces by 13 and 35% of overweight/obesity and type 2 diabetes [3], respectively. Moreover, breastfeeding is positively associated with intelligence in later life [4, 5].

The World Health Organization (WHO) and the United Nations Children’s Fund (UNICEF) jointly recommend that breastfeeding should be initiated within the first hour after birth, continued exclusively for the first 6 months and then continued with safe and adequate complementary foods until the age of 2 years or older [6]. Early initiation of breastfeeding (EIBF) and exclusive breastfeeding (EBF) are considered as “sentinel indicators” for facility-based monitoring of clinical practices related to breastfeeding [7].

In 2012, the World Health Assembly adopted a series of nutrition-related goals, one of which is to “increase the rate of exclusive breastfeeding in the first 6 months to at least 50% by 2025” [8]. Breastfeeding can also be linked to eight of the Sustainable Development Goals (SDGs) [7]. However, recent data showed that worldwide, only 42.4% of infants initiated breastfeeding early, 40.7% were exclusively breastfed within the first 6 months, and 45.1% continued breastfeeding until 2 years [9].

Basic data are crucial for evaluating the breastfeeding practice in achieving these goals. However, even as recently as 2018, China relied on out-of-date data from the China National Health Services Survey 2008 and 2013 Nutrition and Health Surveillance System to report statistics to the UNICEF Global Database on Infant and Young Child Feeding [10]. Other domestic data on breastfeeding practices in China come from scattered cross-sectional surveys [11–14]. Moreover, two national surveys report obviously different prevalence of EBF in China in the same year [15, 16]. With the implementation of health promotion actions and education, an increasing number of pregnant women have recognized the benefits of breastfeeding and are willing to follow recommended practices. Current status of breastfeeding practices in China, especially in different parts of China, and how those practices have changed over time are of great interest. A recent review, which updates the changes of breastfeeding in China in the past decade, indicates the improvement of “any breastfeeding” prevalence, while the EBF prevalence is still low [17]. In addition, it acknowledges large gaps existed among studies conducted in different areas. Many factors contribute to these gaps, such as study methods, study populations, educational and economic background, residential area [17].

Therefore, long time series studies which covering different areas are needed to give an accurate evaluation on the progress in breastfeeding practice in China. Furthermore, as home to the world’s second largest population of infants and children, China may influence breastfeeding practices around the world. With large geographic disparities in population size and economic development, China’s experiences may also help clarify what influences practices in other countries alike.

This study used data from a long-term national surveillance system to analyze the annual prevalence of EIBF and EBF in China and in different parts of China, and to investigate the progress of breastfeeding and bottlenecks in driving better breastfeeding practice.

## Methods

### Study design

This study used data from the Under 5 Child Nutrition and Health Surveillance System (U5CNHSS), a component of the National Maternal and Child Health Surveillance System (NMCHSS) in China [18]. Although the surveillance system was running from October 1, 2011, data quality was erratic until 2013. Therefore, we used data from 2013 to 2018 in the present study. The U5CNHSS contains data on about 140,000 children under 5 years each year, including almost 40,000 infants under 6 months. The U5CNHSS dynamically collected information on growth and development, nutrition and health status, and impact factors of nutrition and health at 1, 3, 6, 8, 12, 18, 24 and 30 months, as well as at 3 and 4 years for each child. After multi-stage stratified cluster random sampling of the NMCHSS, we selected 80 of the 334 districts/counties in mainland China. First, all districts/counties in China were divided into four levels (urban, eastern rural, central rural and western rural areas) and ranked by total population. The reported rate of underweight among children under 5 years in the four levels were used to calculate the sample size in each level. We imposed a requirement of data from at least 2000 children in each district/county in order to compensate for loss of subjects due to mobility and other causes. Then the number of districts/counties in each level were decided. Second, four levels were further divided into subgroups based on the number of districts/counties. Third, 334 districts/counties from NMCHSS were assigned to each subgroup. Then one district/county was randomly selected from each subgroup. Fourth, four townships (communities/sub-districts) in each selected district/county were systematically sampled based on total population. Finally, villages/committees randomly selected until reaching the required minimal sample size.

In one part of this study, trends in the two breastfeeding indicators over time were analyzed based on the cross-sectional survey data. In the second part of this study, prevalence of exclusive breastfeeding over time was analyzed based on longitudinal cohort data.

### Study population

Analysis of EIBF trends involved data for infants born between January 1, 2013 and December 31, 2018 by year. Analysis of EBF trends involved data for infants who were surveyed between September 1 and September 30 in each year of the study period and who were no older than 180 days at the surveys. Longitudinal analysis of EBF prevalence involved data for infants who participated in all three visits at 1, 3 and 6 months and who were no older than 180 days at the 6-month visit. The study flow chart was shown in the Supplementary Figure 1.

### Data collection

Staff collecting data were trained before the surveillance. Unified protocol and study forms were used for each surveillance site. Briefly, village or committee doctors were required to register all newborns and under-5 children within their areas of responsibility. They conducted family visits of newborns, and notified children for health visits. Doctors at township or community health care centers assembled a health profile for each child and were responsible for health visits. During the health visits, the children’s weight and length/height were measured, and they underwent a basic physical exam and developmental assessment, and their hemoglobin were tested (once every year). During the visits, parents were asked to complete a structured questionnaire collecting demographic information about the household, the child’s diseases, and feeding practices. Data were entered into the network reporting system within 1 month after each visit, and data were subjected to a quality control process that was audited level-by-level.

EIBF and EBF were defined according to the WHO [19]. We calculated the indicators for each year during the study period. EIBF was defined as the proportion of infants born in the year who were put to the breast within 1 hour of birth. This was based on recall by infants’ mothers at the first visit, who were asked “How soon after birth was your baby be first put to the breast? [1=never, 2=within 1 hour (immediately), 3=1-23 hours, 4=the second day or later, 5=unknown]”.

EBF was defined as the proportion of infants 0–5 months old who were fed exclusively with breast milk, including breastfeeding by a wet nurse and feeding expressed breast milk, as well as administration of any necessary medications, vitamins, or oral rehydration solution. This indicator was based on recall of the previous day (i.e. 24 h before) by mothers at each visit, who were asked “Did your infant receive breast milk yesterday (i.e. 24 hours ago)?” If they responded “yes”, then they were asked, “Which kind of liquid food did your infant receive yesterday?” and allowed to choose from preset responses (1 = water, 2 = sweet water/juice/other liquid food, 3 = milk powder/milk/goat’s milk, 4 = formula milk powder, 5 = none of above, 6 = unknown). The women who answered “yes” were also asked, “Did your infant receive solid/semi-solid/soft food yesterday (such as rice flour, paste, cooked rice, steamed bread, etc)?” and allowed to choose from preset responses (1 = yes, 2 = no, 3 = unknown). “Exclusive breastfeeding” was defined as the feeding of infants 0–5 months old with breast milk but neither liquid food listed in the questionnaire nor solid/semi-solid/soft food during the previous 24 h.

### Quality control

The village or community doctors checked the list of newborns and under-5 children monthly to make sure that each child participated in health visits on time. Health workers in the township or community health care centers cross-checked their lists of children who finished health visits with the lists of the village or community doctors, and they also checked the completeness of data collection forms. Health workers at the levels of county/district, prefecture, province, and nation would sample 2–3 surveillance sites semi-annually or annually for quality control concerning work flow, measuring methods, instrument adjustment records and questionnaire data quality.

### Statistical analysis

In the analysis, mainland China was divided into three regions (i.e., East, Central and West) based on economic development level as per the surveillance system, and we divided the country into rural and urban areas based on classification by the National Bureau of Statistics of China [20].

The prevalence of the two breastfeeding indicators with 95% confidence intervals (95% CIs) were calculated at the national and subnational levels. The Cochran-Armitage trend test [21, 22] was used to explore trends in the two breastfeeding indicators over time. The annual percent of change (APC) in prevalence of the two breastfeeding indicators at the national and subnational levels were estimated, together with 95% CIs, using log-linear regression [log(*δ*_*b*_) = log(*δ*_*a*_) + (*t*_*b*_ *− t*_*a*_)log(*θ*)], followed by *exp* transformation [APC = exp. (log(*θ*))-1) × 100] [23].

All analyses were performed using SAS 9.4 (SAS Institute, Cary, NC, USA). Statistical significance was assessed by two-tailed tests at an alpha level of 0.05.

## Results

### Time trends analysis on the prevalence of EIBF

**Fig. 1:**
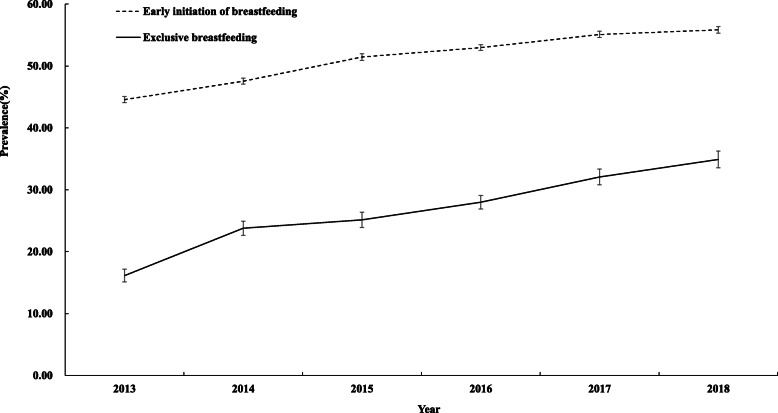
Trends in prevalence of early initiation of breastfeeding (dotted) and exclusive breastfeeding (full) in China, 2013–2018. The percent of infants who early initiated breastfeeding and who were exclusively breastfed were reported as the prevalence (%). Error bars indicated 95% confidence intervals, which were based on binomial proportion estimation

222,641 infants were included in the EIBF trends analysis. As shown in Fig. 1, the prevalence of EIBF increased from 44.57% (95% CI: 44.07, 45.07) in 2013 to 55.84% (95% CI: 55.29, 56.38) in 2018 (P_trend_ < 0.001). The APC was 4.67% (95% CI: 3.51, 5.85).

**Fig. 2:**
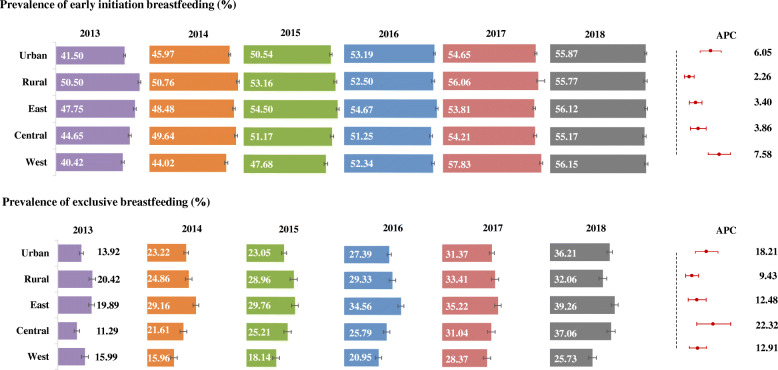
Prevalence of early initiation of breastfeeding (*upper panel*) and exclusive breastfeeding (*lower panel*) in different parts of China, 2013–2017. The percent of infants who early initiated breastfeeding and who were exclusively breastfed in different parts of China were reported as the prevalence (%). Error bars of prevalence indicated 95% confidence intervals (CI), which were based on binomial proportion estimation. APC stood for annual percent change

Prevalence of EIBF increased significantly from 2013 to 2018 in both urban and rural areas (P_trend_ < 0.001), with the APC greater in urban areas (6.05%; 95% CI: 4.22, 7.92) than in rural areas (2.26%; 95% CI: 1.40, 3.12). Among the three regions of China, the West showed the greatest APC (7.58%; 95% CI: 5.63, 9.56), which reached 56.15% (95% CI: 55.10, 57.20) in 2018 (Fig. 2).

### Time trends analysis on the prevalence of EBF

30,876 infants were included in the EBF trends analysis. The prevalence of EBF increased from 16.14% (95% CI: 15.10, 17.18) in 2013 to 34.90% (95% CI: 33.54, 36.26) in 2018 (P_trend_ < 0.001), with an APC of 14.90% (95% CI: 9.97, 20.04) (Fig. 1).

Similarly, prevalence of EBF increased rapidly from 2013 to 2018 in all areas across China, with greater APC in urban areas (18.21%; 95% CI: 11.53, 25.29) than in rural areas (9.43%; 95% CI: 5.52, 13.49). The highest EBF prevalence was observed in the East (39.26%; 95% CI: 37.14, 41.38 in 2018), but the Central area showed the highest APC of 22.32% (95% CI: 12.36, 33.15) (Fig. 2).

### Longitudinal analysis on the prevalence of EBF

A total number of 15,417 infants were included in the subsequent analysis. The prevalence of EBF decreased with increasing age within the first 6 months, especially after 3 months. However, increasing trends were observed from 2013 to 2018 at each visit and among all areas (Table 1**)**. EBF prevalence at 6-month visit increased rapidly from 3.25% (95% CI: 2.59, 3.91) in 2013 to 10.50% (95% CI: 9.01, 11.99) in 2018, with an APC of 24.22% (95% CI: 19.12, 29.54). In 2018, EBF prevalence was 9.91% (95% CI: 8.14, 11.67) in urban areas, higher than the 11.74% (95% CI: 9.00, 14.49) in rural areas. Similarly, the APC was higher in urban areas (28.82%; 95% CI: 15.93, 43.15 v.s. 20.89%; 95% CI: 13.95, 28.26). The least progress in EBF was observed in the West, where its prevalence in 2018 was 32.47% (95% CI: 27.55, 37.39) at 1-month visit, 22.99% (95% CI: 18.57, 27.41) at 3-month visit and 5.17% (95% CI: 2.85, 7.50) at 6-month visit.

**Table 1:** Prevalence of exclusive breastfeeding among infants at 1, 3 and 6 months’ visit in China, 2013 to 2018^a^

Group	Year of birth	N	1-month visit, N (%; 95% CI)	3-month visit, N (%; 95% CI)	6-month visit, N (%; 95% CI)
Total	2013	2767	581 (21.00; 19.48, 22.52)	319 (11.53; 10.34, 12.72)	90 (3.25; 2.59, 3.91)
	2014	3093	854 (27.61; 26.04, 29.19)	536 (17.33; 16.00, 18.66)	159 (5.14; 4.36, 5.92)
	2015	2684	834 (31.07; 29.32, 32.82)	548 (20.42; 18.89, 21.94)	157 (5.85; 4.96, 6.74)
	2016	3081	927 (30.09; 28.47, 31.71)	678 (22.01; 20.54, 23.47)	221 (7.17; 6.26, 8.08)
	2017	2164	691 (31.93; 29.97, 33.90)	484 (22.37; 20.61, 24.12)	185 (8.55; 7.37, 9.73)
	2018	1628	680 (41.77; 39.37, 44.16)	507 (31.14; 28.89, 33.39)	171 (10.50; 9.01, 11.99)
	APC	–	11.60 (6.63, 16.81)	18.05 (11.47, 25.03)	24.22 (19.12, 29.54)
Urban	2013	1577	287 (18.20; 16.29, 20.10)	165 (10.46; 8.95, 11.97)	37 (2.35; 1.60, 3.09)
	2014	1836	516 (28.10; 26.05, 30.16)	330 (17.97; 16.22, 19.73)	101 (5.50; 4.46, 6.54)
	2015	1653	490 (29.64; 27.44, 31.84)	323 (19.54; 17.63, 21.45)	93 (5.63; 4.52, 6.74)
	2016	2025	546 (26.96; 25.03, 28.90)	424 (20.94; 19.17, 22.71)	132 (6.52; 5.44, 7.59)
	2017	1372	399 (29.08; 26.68, 31.48)	287 (20.92; 18.77, 23.07)	125 (9.11; 7.59, 10.63)
	2018	1100	452 (41.09; 38.18, 44.00)	365 (33.18; 30.40, 35.96)	109 (9.91; 8.14, 11.67)
	APC	–	12.36 (4.30, 21.05)	19.70 (10.35, 29.85)	28.82 (15.93, 43.15)
Rural	2013	1190	294 (24.71; 22.26, 27.16)	154 (12.94; 11.03, 14.85)	53 (4.45; 3.28, 5.63)
	2014	1257	338 (26.89; 24.44, 29.34)	206 (16.39; 14.34, 18.43)	58 (4.61; 3.45, 5.77)
	2015	1031	344 (33.37; 30.49, 36.24)	225 (21.82; 19.30, 24.34)	64 (6.21; 4.73, 7.68)
	2016	1056	381 (36.08; 33.18, 38.98)	254 (24.05; 21.48, 26.63)	89 (8.43; 6.75, 10.10)
	2017	792	292 (36.87; 33.51, 40.23)	197 (24.87; 21.86, 27.88)	60 (7.58; 5.73, 9.42)
	2018	528	228 (43.18; 38.96, 47.41)	142 (26.89; 23.11, 30.68)	62 (11.74; 9.00, 14.49)
	APC	–	11.52 (8.91, 14.20)	15.38 (9.83, 21.20)	20.89 (13.95, 28.26)
East	2013	1274	259 (20.33; 18.12, 22.54)	178 (13.97; 12.07, 15.88)	65 (5.10; 3.89, 6.31)
	2014	1549	486 (31.38; 29.06, 33.69)	337 (21.76; 19.70, 23.81)	112 (7.23; 5.94, 8.52)
	2015	1241	419 (33.76; 31.13, 36.39)	276 (22.24; 19.93, 24.55)	100 (8.06; 6.54, 9.57)
	2016	1499	521 (34.76; 32.35, 37.17)	385 (25.68; 23.47, 27.90)	142 (9.47; 7.99, 10.96)
	2017	1073	388 (36.16; 33.29, 39.04)	279 (26.00; 23.38, 28.63)	118 (11.00; 9.13, 12.87)
	2018	760	304 (40.00; 36.52, 43.48)	249 (32.76; 29.43, 36.10)	88 (11.58; 9.30, 13.85)
	APC	–	11.59 (4.88, 18.73)	15.16 (8.92, 21.75)	17.07 (12.58, 21.74)
Central	2013	676	102 (15.09; 12.39, 17.79)	41 (6.07; 4.27, 7.86)	8 (1.18; 0.37, 2.00)
	2014	760	171 (22.50; 19.53, 25.47)	98 (12.89; 10.51, 15.28)	30 (3.95; 2.56, 5.33)
	2015	855	236 (27.70; 24.69, 30.70)	159 (18.66; 16.05, 21.28)	32 (3.76; 2.48, 5.03)
	2016	906	245 (27.04; 24.15, 29.93)	178 (19.65; 17.06, 22.23)	57 (6.29; 4.71, 7.87)
	2017	562	165 (29.36; 25.59, 33.12)	108 (19.22; 15.96, 22.47)	43 (7.65; 5.45, 9.85)
	2018	520	263 (50.58; 46.28, 54.87)	178 (34.23; 30.15, 38.31)	65 (12.50; 9.66, 15.34)
	APC	–	21.53 (12.54, 31.24)	32.71 (17.75, 49.57)	50.43 (31.07, 72.65)
West	2013	817	220 (26.93; 23.89, 29.97)	100 (12.24; 9.99, 14.49)	17 (2.08; 1.10, 3.06)
	2014	784	197 (25.13; 22.09, 28.16)	101 (12.88; 10.54, 15.23)	17 (2.17; 1.15, 3.19)
	2015	591	179 (30.29; 26.58, 33.99)	113 (19.12; 15.95, 22.29)	25 (4.23; 2.61, 5.85)
	2016	676	161 (23.82; 20.61, 27.03)	115 (17.01; 14.18, 19.84)	22 (3.25; 1.92, 4.59)
	2017	529	138 (26.09; 22.35, 29.83)	97 (18.34; 15.04, 21.63)	24 (4.54; 2.76, 6.31)
	2018	348	113 (32.47; 27.55, 37.39)	80 (22.99; 18.57, 27.41)	18 (5.17; 2.85, 7.50)
	APC	–	2.34 (−3.34, 8.34)	12.41 (6.45, 18.70)	20.43 (10.08, 31.75)

*APC* average annual percent change, *CI* confidence interval, *N* number of infants

^a^Infants who attended all three visits (i.e., 1 month, 3 month, and 6 month) and were younger than 180 days old were included in the analysis

## Discussion

This study reported increasing trends of the two sentinel indicators of breastfeeding practices in China from 2013 to 2018. Urban areas showed greater progress than rural areas. Maternal and child health is highly valued by the Chinese government, which has implemented national initiatives to promote breastfeeding practices. In 1994, the Chinese government issued a law on maternal and infant health care [24], followed by numerous measures to implement the law [25]. Under these regulations, healthcare facilities are required to give guidance on maternal breastfeeding, and prohibit the promotion of breast milk substitutes. Since the 1990s, China has promoted breastfeeding practices by evaluating hospitals for the status of baby-friendly hospitals (BFHs). Then guidelines were released for management and supervision of BFHs [26] as well as standards for the review of BFHs [27]. After rigorous evaluation, the National Health Commission announced 7036 BFHs [27]. In spite of these efforts, Guo and colleagues found no improvement on EBF prevalence in China from 2003 to 2010 [28]. In 2012, the Ministry of Health launched maternal and infant health literacy - basic knowledge and skills (trial) [29]. In the same year, the China Alliance for Breastfeeding Action was established, which released China Promoting System for Breastfeeding Planning [30]. Furthermore, diverse activities were organized every year during World Breastfeeding Week. All these policies and actions have fostered a positive environment for breastfeeding in China. However, the EBF prevalence according to the Chinese National Nutrition and Health Survey was even lower in 2013 than in 2008 [16].

In the present study, the prevalence of EIBF increased from 44.57% in 2013 to 55.84% in 2018, which seems slightly higher than the global average (42.4%) [9]. However, this prevalence is still lower than that in 33 of 131 countries (25.19%) in the 2018 UNICEF Global Database on Infant and Young Child Feeding [10]. Furthermore, an increase of only 1.34% was observed between 2017 and 2018, which means the growth was slowing. We noticed that with a higher APC in urban areas, EIBF prevalence in urban areas reached the same level as the rural areas until 2018. Similarly, the three regions of China showed minimal differences in 2018. The Global Nutrition Report for the same year also showed similar EIBF prevalence between urban and rural areas, and between the lowest and highest quintile areas [9].

Early initiation of breastfeeding can reduce both neonatal and early infant mortality [31–33]. Many factors are associated with early breastfeeding initiation. As reported in other studies, some mothers thought colostrum was dirty and bad for baby, and they discarding colostrum [34]. This could also be seen in some parts of China, especially the west and rural China. Caesarean section and higher education may negatively affect the breastfeeding practice [35]. This could partly explain the variations among different regions across China. But specific surveys are needed to identify the exact factors behind these differences. Studies showed skin-to-skin contact and health education were positively associated with prevalence of EIBF [36–38]. A recent review from the US concluded that baby-friendly practices can help increase prevalence of EIBF and EBF [39]. It seems supporting the construction of BFHs and promoting their role may help improve breastfeeding practices. However, it has been reported that only 12% of births in China occur in BFHs [40]. Therefore, more studies are needed to compare breastfeeding practices between BFHs and non-BFHs in China.

EBF prevalence may depend strongly on the age of the study population: in our study, it decreased with increasing age within the baby’s first 6 months. Due to the relatively young age of our sample, we may overestimate the prevalence of EBF in this study. Throughout the study period, EBF prevalence in China has progressed in recent years (with the mean ages of 71.8, 70.1, 73.2, 71.1, 71.3 and 69.8 days from 2013 to 2018). The urban areas progressed a lot, with even a reversed higher EBF prevalence than rural areas in 2018.

There were some obstacles in promoting better breastfeeding practice in China. The first obstacle is the unbalanced progress in breastfeeding practice among different areas in China, reflected in large differences in EBF prevalence. The rural and West China should be the key areas in the future actions. The second obstacle is the high proportion of births by cesarean section in China [41], which shows a negative correlation with breastfeeding prevalence [42] and delayed breastfeeding initiation [43]. Our observation of upward trends in EIBF and EBF prevalence gives cause for hope. These encouraging trends may reflect greater awareness of breastfeeding among healthcare professionals and mothers. Another cause for optimism is that a recent hospital-based study reported a decline in cesarean section rates in China from 2012 to 2016 [44]. The third obstacle is the increased participation of women in the labor force and employment away from home without sufficient maternity protection, which may shorten the period of exclusive breastfeeding. Paid breastfeeding breaks of at least 6 months are positively associated with an increase in EBF prevalence [45]. In 2016, China implemented the “Comprehensive Two Child” policy, leading 31 provinces, autonomous regions and municipalities to prolong paid maternity leave from 98 to at least 128 days [46]. Since that year, we observed relatively rapid growth in EBF prevalence at 3 months after birth, especially in urban areas and East China. Moreover, APC also increased in most areas at 6 months. Thus, further extension of paid maternity leave might be a positive factor on promoting EBF practice. The longitudinal analysis showed a sharp decrease in EBF prevalence between 3 and 6 months of age, which is a key period of improvement for better breastfeeding practice in the future. The fourth obstacle is the marketing of infant formula. In 1995, the Chinese government adopted the Code on Marketing of Breast Milk Substitutes [47], but infant formula sales continued to grow rapidly in the country [48]. The implementing of the Code has been recognized as a critical intervention to create an enabling environment for breastfeeding [49]. However, the WHO has classified China as a country with “few provisions in law” in response to the Code [50]. Even if, the Chinese government abandoned the Code in favor of a “Notice Further Regulating the Promotion and Marketing of Breast Milk Substitutes” at the end of 2017. The continued growth of formula sales for infants aged 0 to 5 months in China [48] highlights the need for the government and non-governmental organizations to further regulate marketing of breast milk substitutes.

Our analyses are limited by the fact that the U5CNHSS data are available only for certain time points. In addition, many infants in the U5CNHSS were excluded from our study because their “6-month visit” occurred later than 180 days after birth. This leads to more young infants in our study population, which increases the risk of overestimating EBF and EIBF prevalence.

## Conclusion

Breastfeeding practices in China are improving in recent years, though with disparities between urban and rural areas and among three geographic regions. More study on the barriers and facilitators to breastfeeding practice in China by urban and rural areas and geographical regions are needed. We hope the government, health workers and social forces can join efforts to protect and promote breastfeeding in China to achieve near- and long-term goals for child health.

## Supplementary Information

**Additional file 1: Supplementary Figure 1.** The flow chart of the participants. This figure illustrates the flow of inclusion and exclusion of subjects in the analysis.

## Data Availability

This study used data from the U5CNHSS. This system was co-established by the National Health Commission of the People’s Republic of China and Sichuan University, and it is owned by the National Health Commission of the People’s Republic of China. The researchers did not obtain consent to publicly share the data. The non-identifiable dataset is available upon request to interested researchers. For data requests, please contact the Department of Science and Technology of West China Second University Hospital, Sichuan University, at: fu2yuankjb@163.com.

## Abbreviations

APCs: Annual percent of changes
CIs: Confidence intervals
EIBF: Early initiation of breastfeeding
EBF: Exclusive breastfeeding
NMCHSS: National Maternal and Child Health Surveillance System
SDGs: Sustainable Development Goals
UNICEF: United Nations Children’s Fund
U5CNHSS: Under 5 Child Nutrition and Health Surveillance System of China
WHO: World Health Organization
