# Supplementary material for: Breastfeeding practice in China from 2013 to 2018: a study from a national dynamic follow-up surveillance
Source: BMC Public Health. 2021 Feb 10;21:329. doi: 10.1186/s12889-021-10211-2 (PMC7874457; doi:10.1186/s12889-021-10211-2)
Supplement: Supplementary file 1 — Additional file 1: Supplementary Figure 1. The flow chart of the participants. This figure illustrates the flow of inclusion and exclusion of subjects in the analysis. [file 12889_2021_10211_MOESM1_ESM.pptx]

## Slide 1
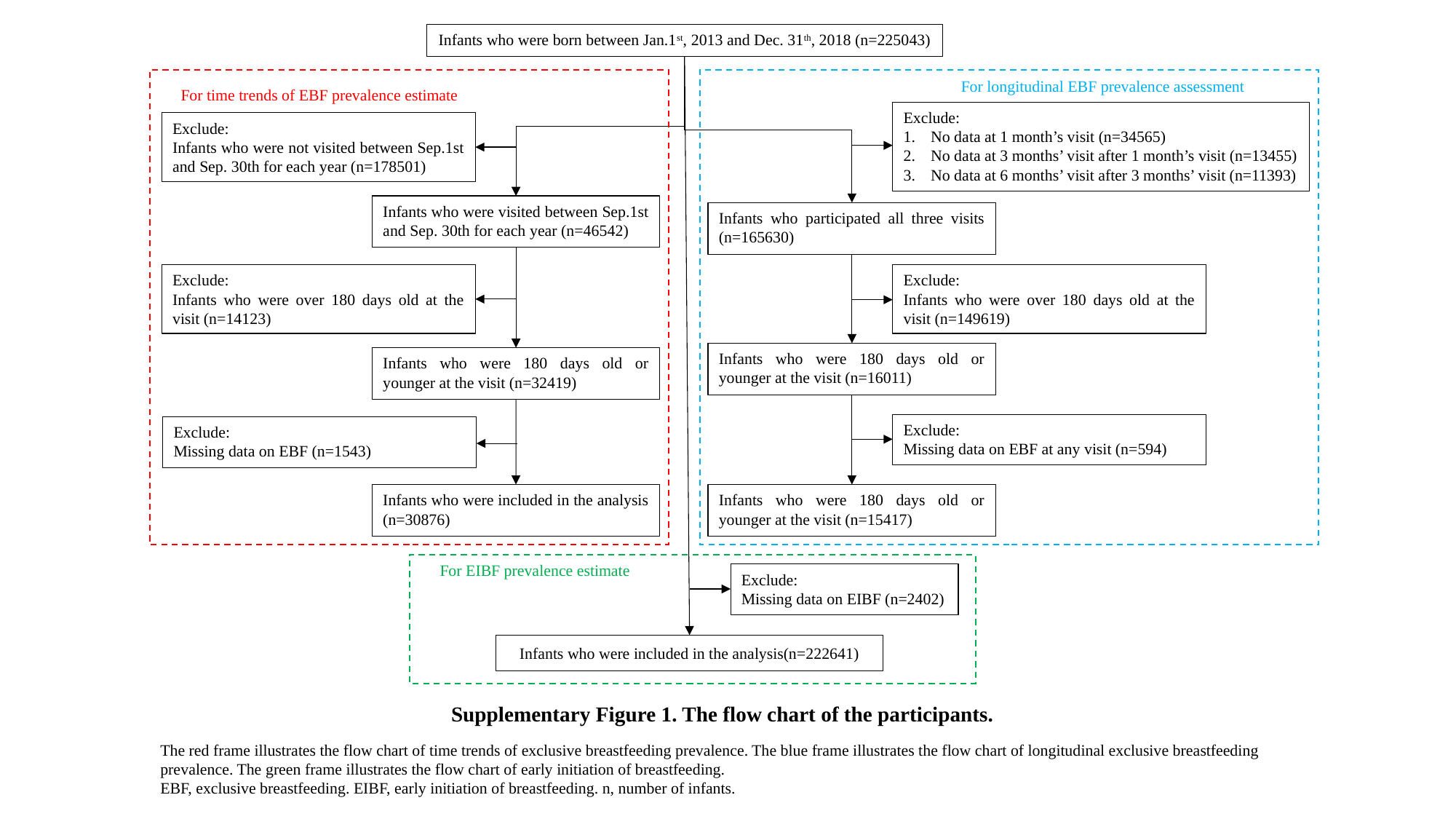

Infants who were born between Jan.1st, 2013 and Dec. 31th, 2018 (n=225043)
For longitudinal EBF prevalence assessment
For time trends of EBF prevalence estimate
Exclude:
No data at 1 month’s visit (n=34565)
No data at 3 months’ visit after 1 month’s visit (n=13455)
No data at 6 months’ visit after 3 months’ visit (n=11393)
Exclude:
Infants who were not visited between Sep.1st and Sep. 30th for each year (n=178501)
Infants who were visited between Sep.1st and Sep. 30th for each year (n=46542)
Infants who participated all three visits (n=165630)
Exclude:
Infants who were over 180 days old at the visit (n=14123)
Exclude:
Infants who were over 180 days old at the visit (n=149619)
Infants who were 180 days old or younger at the visit (n=16011)
Infants who were 180 days old or younger at the visit (n=32419)
Exclude:
Missing data on EBF at any visit (n=594)
Exclude:
Missing data on EBF (n=1543)
Infants who were included in the analysis (n=30876)
Infants who were 180 days old or younger at the visit (n=15417)
For EIBF prevalence estimate
Exclude:
Missing data on EIBF (n=2402)
Infants who were included in the analysis(n=222641)
Supplementary Figure 1. The flow chart of the participants.
The red frame illustrates the flow chart of time trends of exclusive breastfeeding prevalence. The blue frame illustrates the flow chart of longitudinal exclusive breastfeeding prevalence. The green frame illustrates the flow chart of early initiation of breastfeeding.
EBF, exclusive breastfeeding. EIBF, early initiation of breastfeeding. n, number of infants.
